# Near-Infrared Rewritable, Non-Volatile Subwavelength Absorber Based on Chalcogenide Phase Change Materials

**DOI:** 10.3390/nano10061222

**Published:** 2020-06-23

**Authors:** Jianfa Zhang, Yiqiong Zhang, Qilin Hong, Wei Xu, Zhihong Zhu, Xiaodong Yuan

**Affiliations:** College of Advanced Interdisciplinary Studies, National University of Defense Technology, Changsha 410073, China; zyqfamily@sina.com (Y.Z.); qlhong95@126.com (Q.H.); weixu08a@163.com (W.X.); zzhwcx@163.com (Z.Z.)

**Keywords:** absorber, rewritable, near-infrared, chalcogenide phase change materials

## Abstract

Chalcogenide phase change materials enable the realization of novel, non-volatile, switchable electronic and photonic devices. In this paper, we propose a type of rewritable, non-volatile near infrared subwavelength absorber based on chalcogenide phase change materials. Our numerical simulations show that nearly perfect absorption more than 0.99 can be realized in the written state while the absorption of as-deposited or erased state is lower than 0.15 in the studied spectral range, leading to high contrast ratio of reflection more than 20 dB. Continuous tuning of the absorption spectra can be realized not only by varying the geometric parameters of the absorber but also by changing the crystallization ratio of the switched Ge${}_{2}$Sb${}_{2}$Te${}_{5}$ (GST). The proposed device may find widespread applications in optical modulation, beam steering and so on.

## 1. Introduction

Subwavelength photonic devices, represented by plamsonic devices and metamaterials, have attracted intensive research in the past decade. The flexibility in design and the unprecedented capabilities of controlling the amplitude, phase and polarization of light enable the realization of a variety of functionalities by them, leading to a new generation of flat, minimized and highly efficient nanophotonic components and devices [1]. The functionalities of traditional nanophotonic devices are fixed once fabricated, which hinders their applications in many situations where real-time tuning are required and leads to the development of tunable nanopotonic devices. One of the most effective methods is combining the nanophotonic devices with active materials whose optical properties can be changed via external stimuli [2].

Non-volatile chalcogenide phase change materials, with unique properties such as drastic optical/electrical contrasts, good thermal stability, fast switching speed and many achievable rewriting cycles, are widely exploited in rewritable optical disk storage technology and non-volatile electronic memories [3,4,5,6,7]. Ge${}_{2}$Sb${}_{2}$Te${}_{5}$ (GST) is one of the most well-known chalcogenide phase change materials. It has a crystallization temperature Tc of about 160 ${}^{{}^{\circ}}$C and a melting temperature Tm of about 600 ${}^{{}^{\circ}}$C. GST has been extensively proven as a platform for fast, robust, reproducible, non-volatile phase switching [8,9]. The novel optical properties, along with its relative mature industry applications, make GST a very attractive choice for the development of tunable optical devices [10,11]. A variety of optically tunable nanophotonic devices and functionalities based on GST have been demonstrated, such as tunable metamaterials [12,13,14,15], dynamic color display [16,17,18], beam steering [19,20], thermal emission [19,21], scattering [22,23,24,25] and polarization [26,27] control.

Optical absorption plays a key role in many optical and optoelectronic devices. Considerable research efforts have been put into the development of subwavelength perfect absorbers in the past few years, particularly plasmonic and metamaterial absorbers [28,29]. Tunable perfect absorbers based on GST have been theoretically and experimentally studied in both near and mid-infared ranges [30,31,32,33,34,35,36,37]. Most of them employ GST as a space layer (or part of the space layer) sandwiched between the top metallic nanostructures and the bottom reflective metal layer. A few others use nanopatterned GST as Mie resonators [38]. In these designs, GST is switched as a whole. The abilities to write, erase and rewrite two-dimensional binary or grayscale functional patterns into a nanoscale film of GST by inducing a local refractive-index-changing phase transition have not been fully explored [15,39]. In this paper, we propose a type of rewritable, non-volatile and inherently flat subwavelength absorbers with GST. Numerical simulations show that nearly perfect absorption more than 0.99 can be realized at the telecom wavelength range.

## 2. Results and Discussion

**Figure 1:**
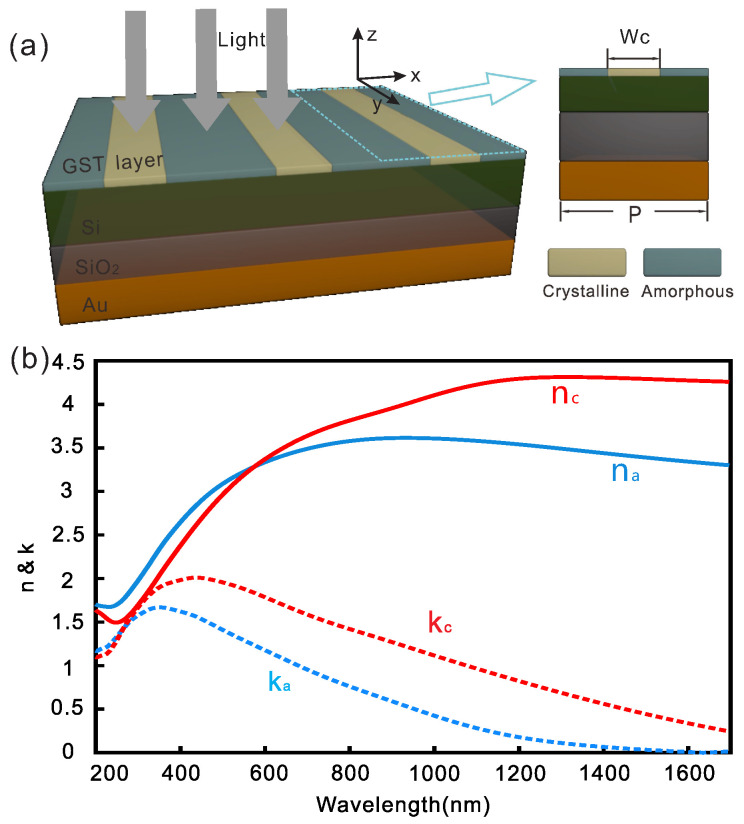
Rewritable subwavelength absorber based on Ge${}_{2}$Sb${}_{2}$Te${}_{5}$ (GST). (**a**) Schematic of a rewritable near infrared absorber. The x-polarized light impinges on the top side of the structure at normal incidence. (**b**) Measured optical constants of GST at crystalline and amorphous states [12].

Figure 1a is the schematic of the proposed subwavelength absorber. From the top to the bottom is a thin GST film, a silicon (Si) waveguide layer, an silicon dioxide (SiO${}_{2}$) insulator layer and a reflective gold layer. The thicknesses of GST, Si, SiO${}_{2}$ and gold layers are 40, 190, 255 and 200 nm, respectively. The top GST layer can be deposited by sputtering and it is generally in the amorphous phase in the as-deposited state. It has been demonstrated that light or focused ion beam-induced grayscale phase transition can be achieved in an extremely small volume of GST [15,39] and the phase change of GST will lead to a large change of refractive index, especially in the infrared range [40]. After part of the GST layer (see the grating in Figure 1) is “written” to the crystalline state, the periodical distributions of amorphous GST (a-GST) and crystalline GST (c-GST) form a refractive index grating at the subwavelength scale. The refractive grating can then be used to scatter and couple free-space light into the underneath waveguide layer. This forms a guided-mode resonance waveguide grating [41]. The bottom gold layer works as a back mirror to block the transmission and enhance the absorption of the absorber. The period of the gratings is fixed at *P* = 765 nm in this paper.

The numerical simulations are conducted using a fully three-dimensional finite element technique (COMSOL Multiphysics, Electromagnetic Waves, Frequency Domain interface in Wave Optics Module). The simulations are conducted in 2D since our structure is uniform in the *y*-direction (see Figure 1a). Periodic Floquet boundary conditions are used in the *x*-direction while port boundary conditions are used at the top and bottom of the modeling area in *z*-direction. A maximum mesh size of 10 nm is used in the thin GST area while a maximum mesh size of 50 nm is used in other areas to ensure the simulation accuracy. In simulations, Si and SiO${}_{2}$ are assumed to be lossless with refractive indices of 3.4 and 1.47, respectively. The permittivity of gold was described by the Drude model with plasma frequency $\omega_{p}=1.37\;\times \;10^{16}\;s^{-1}$ and the damping constant $\omega_{\tau }=1.23\;\times \;10^{14}\;s^{-1}$ which was three times larger than the bulk value considering the increased scattering by surface and grain boundary effects in the thin film. Experimentally measured optical constants of a-GST and c-GST are used in simulations (see Figure 1b).

**Figure 2:**
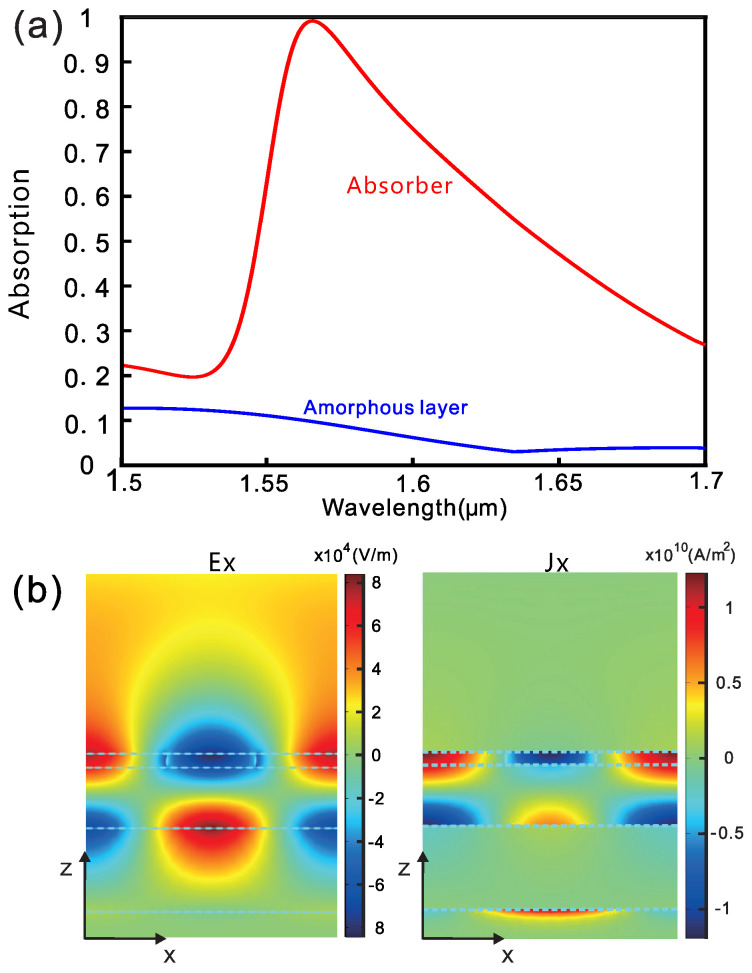
(**a**) Absorption spectra of the subwavelength absorber at “written” and “as-deposited (erased)” (amorphous layer) states. (**b**) Distributions of electrical field (left) and current (right) at the resonance wavelength of 1566 nm. The fully crystallization part of GST covers 35% of the period.

Figure 2a shows the simulated absorption spectra for light impinging at the subwavelength absorber from the top at normal incidence. For the as-deposited GST layer which is in the homogeneous amorphous state, the absorption of the structure depends on the multilayer interference, and it remains low (absorption of A = 0.15 and reflection of R = 0.85) in the studied spectral range and most of the incident light will be reflected. For the absorber where part of the top GST layer is written to the crystalline state and forms a subwavelength grating, a guided mode resonance is excited in the studied spectral range and the absorption spectra dramatically changed. Here the duty ratio is f=35% (the ratio of c-GST, i.e., f=Wc/P where Wc is the width of the crystallized GST). Figure 2b shows the distributions of electric field and current at the resonance wavelength of 1566 nm. The refractive index grating of GST couples the incident free-space light into the underneath Si waveguide where most of the fields are confined. The bottom gold film works as a reflection mirror to block the transmission. With the absorption peak around the telecommunication wavelength, nearly perfect absorption (absorption of A = 0.9916 and reflection of R = 0.0084) can be realized due to critical coupling. This leads to a high contrast ratio of reflection more than 20 dB at the resonance.

**Figure 3:**
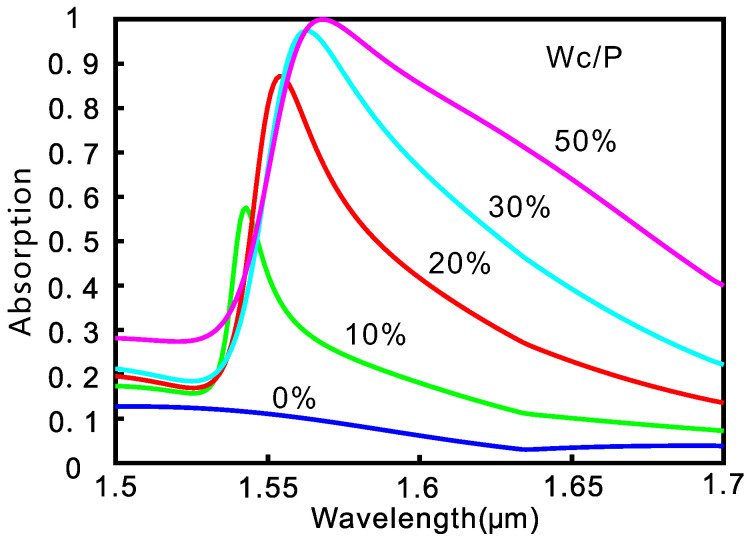
Absorption spectra of the subwavelength absorber with different duty ratio (spatial ratio of the crystallization part).

The excitation of guided mode resonances can be controlled by varying the geometric parameters of the structure, such as the period and duty ratio. As an example, we fix the period of the grating and change the duty ratio. The simulated absroption spectra are shown in Figure 3. With the decrease of the duty ratio, the resonance wavelength blueshifts and the resonance peak drops. When the duty ratio decreases from f=50% to f=10%, the resonance wavelenth shifts from 1568 to 1543 nm and the maximum resonant absorption decreases from 0.9997 to 0.5753. At the same time, the spectral linewidths (full width at half maximum, FWHM) decreases from 132 to 28 nm. The c-GST not only shows an increased refractive index but also an increased absorption coefficient compared to a-GST. So the decrease of duty ratio reduces the effective refractive index and the absorption loss of the GST layer, thereby leading to the blueshift and the increase of the Q-factor of the guided mode resonance. Meanwhile, the absorber realizes perfect absorption at the resonance when critical coupling condition is met, where the coupling rate equals the absorption rate. As the duty ratio is reduced, the device moves away from the critical coupling condition and the absorption peak decreases.

**Figure 4:**
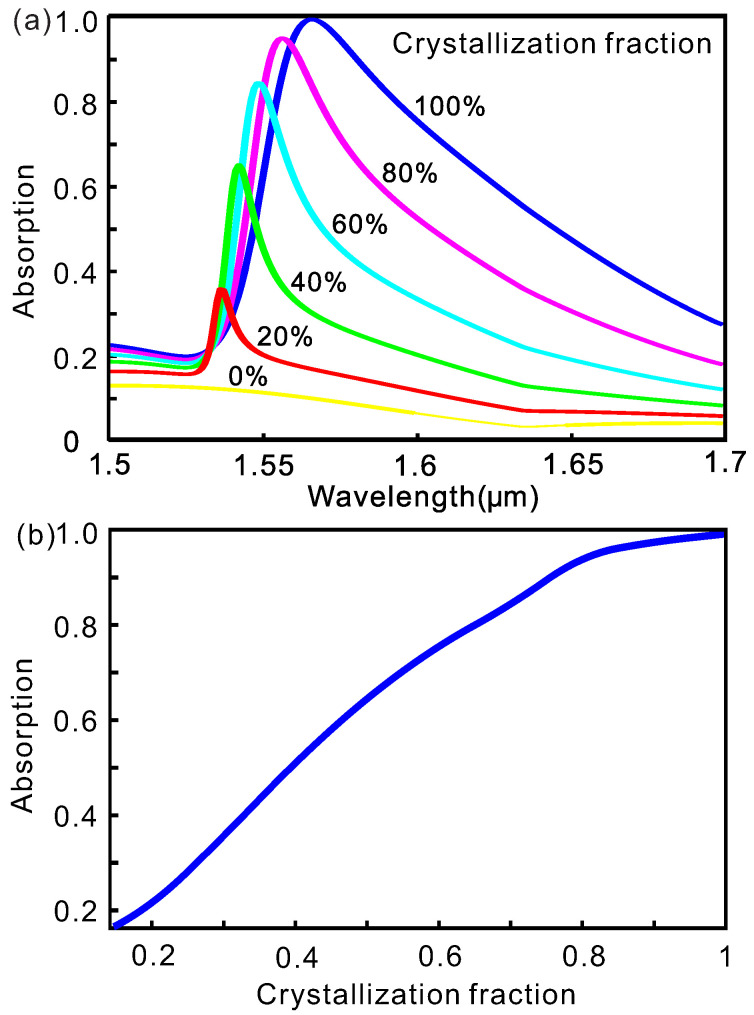
(**a**) Absorption spectra of the subwavelength absorber with different crystallization fraction. (**b**) Variation of maximum resonant absorption as the crystallization fraction of GST increases. The geometric parameters of the GST subwavelength absorber here are the same as those of Figure 2.

The effective dielectric constant of GST with a hybridization of a-GST and c-GST can be calculated using the Lorenz–Lorentz relation,

(1) $$\frac{\varepsilon_{eff}\left(\lambda \right)-1}{\varepsilon_{eff}\left(\lambda \right)+2}=\eta \times \frac{\varepsilon_{c-GST}\left(\lambda \right)-1}{\varepsilon_{c-GST}\left(\lambda \right)+2}+\left(1-\eta \right)\times \frac{\varepsilon_{a-GST}\left(\lambda \right)-1}{\varepsilon_{a-GST}\left(\lambda \right)+2}$$

where $\varepsilon_{c-GST}\left(\lambda \right)$ and $\varepsilon_{a-GST}\left(\lambda \right)$ are the wavelength-dependent dielectric constants of GST in crystalline and amorphous state, respectively, and η is the crystallization fraction of GST. Now we fix the duty ratio of hybrid GST as Wc/P=35% and vary the crystallization fraction of GST η. The optical absorption spectra are shown in Figure 4a. Reducing the crystallization fraction of GST displays a similar influence on the absorption spectra as that of reducing the duty ratio. With the decrease of the crystallization fraction, the resonance wavelength blueshifts and the resonance peak drops. Meanwhile, the resonance becomes sharper due to the reduction of the losses in GST. For example, with a crystallization fraction of 40%, the resonance wavelenth blueshifts to 1542 nm with a FWHM of 25 nm and the maximum resonance drops to 0.6482. The dependence of the maximum resonant absorption on the crystallization fraction is shown in Figure 4b. By switching the GST to the multi-level regime, i.e., metastable semi-crystallized states, we can tune the spectra continuously.

**Figure 5:**
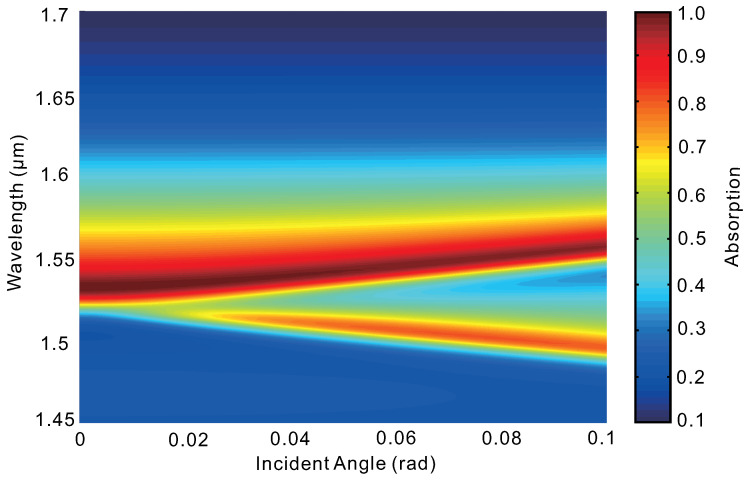
Angular dependence of absorption spectra. Here the parameters of the structure are the same as those of Figure 2.

Figure 5 shows the angular dependence of the absorber. Differently from the metamaterial or plasmonic absorbers based on localized resonances whose dependence on the incident angle is generally weak, the guided mode resonance here is sensitive to the incident angle. This is not difficult to understand, since phase matching plays an important role in the excitation of guided mode resonance. When the incidence light is tilted, an additional absorption peak (resonance) appears in the absorption spectrum. This is because at normal incidence the two resonances are indistinguishable while a non-zero incidence angel removes the degeneracy [41]. The wavelengths of these two resonant absorption peaks shift almost linearly with the incident angle, as it is small. Similar characteristics are typical and have been reported in other guided-mode resonant structures [42]. For applications that are required to work at broad angles, this is a drawback. On the other hand, it could be exploited for applications such as directional thermal emitters [43], optical filters [41] and so on.

## 3. Conclusions

In summary, we have proposed a type of rewritable, non-volatile subwavelength absorber based on phase change material GST. Our numerical simulations show that nearly perfect absorption more than 0.99 can be realized at the written state, and the absorption of the as-deposited or erased state is lower than 0.15 in the studied spectral range, leading to high contrast ratio of reflection more than 20 dB. Continuous tuning of the absorption spectra can be realized not only by varying the geometric parameters of the absorber but also by changing the crystallization ratio of the switched GST. The proposed absorber can be fabricated by thin film deposition technology, and the subwavelength functional patterns may be written, erased and rewritten into the phase-change films with suitably tailored laser pulses [15]. To overcome the diffraction of lasers, focused ion beam induced grayscale phase changing can be used [39]. Another possible method is using electrical pulses (with suitable electrodes) to write and erase the patterns in GST. An etching process can be avoided. This work will promote the research of rewritable nanophotonic devices based on chacolgenide phase change materials, and the proposed absorber may find wide applications from light modulation, beam steering and dynamic display, to optical artificial neural networks and so on.
